# The Link Between Statins and Breast Cancer in Mouse Models: A Systematic Review

**DOI:** 10.7759/cureus.31893

**Published:** 2022-11-25

**Authors:** Raj Watson, Angela Tulk, Jennifer Erdrich

**Affiliations:** 1 Department of Surgery, University of Arizona College of Medicine - Tucson, Tucson, USA

**Keywords:** chemoprevention, metastasis, chemotherapy, anti-cancer, breast cancer, statins

## Abstract

Statins, a class of cholesterol-lowering drugs, have consistently demonstrated pleiotropic effects in both preclinical and clinical studies. Outside of inhibiting the production of cholesterol in cells, statins have shown antineoplastic properties most commonly in breast cancer. Clinical and epidemiological studies, however, are less definitive than preclinical studies regarding statins as potential adjuvant oncologic therapy. Our objective is to summarize mouse model studies that investigate the link between statins and breast cancer using a cancer care continuum framework to provide a clinically relevant picture of the potential use of statins in breast cancer. A systematic review of the PubMed database was performed to identify studies published between January 2007 and July 2022 that investigated the effects of statins on breast cancer prevention, treatment, and survivorship in mouse models. Overall, 58 studies were identified using our search strategy. Based on our inclusion and exclusion criteria, 26 mouse model studies were eligible to be included in our systematic review. In breast cancer mouse models, statins alone and in combination with anti-cancer therapies demonstrate proven antineoplastic effects across the cancer care continuum. The antineoplastic benefit of statins as single agents in mouse model studies helps inform their synergistic benefit that future clinical studies can test. Parameters such as statin timing, dose, and breast cancer subtype are key stepping stones in defining how statins could be used in the treatment of breast cancer.

## Introduction and background

Statins as cholesterol-lowering agents

In the 1970s, Japanese biochemist Akria Endo first discovered molecules that inhibited 3-hydroxy-3-methylglutaryl coenzyme A reductase (HMGCR), an enzyme key to cholesterol biosynthesis. Later, in 1978, Merck Laboratories isolated another HMGCR inhibitor from the fungus *Aspergillus terreus* that would become the first commercial statin, lovastatin. In clinical trials, lovastatin was found to dramatically lower low-density lipoprotein cholesterol (LDL) with few adverse effects. Eventually, lovastatin was approved by the United States Food and Drug Administration in September 1987 and indicated for the treatment and prevention of coronary heart disease, hypercholesterolemia, and adolescents with heterozygous familial hypercholesteremia. Since the approval of lovastatin, six statins have been introduced in the market and it is estimated that more than 200 million people worldwide take a statin [1,2].

Used as first-line treatment against hypercholesteremia and as a preventative treatment for cardiovascular disease (CVD), heart attack, and stroke, statins are a widely prescribed class of drugs with proven safety and efficacy profiles. Statins are competitive antagonists of HMGCR [3-8]. HMGCR is one enzyme in the mevalonate pathway, which is a metabolic cascade responsible for synthesizing cholesterol, an important molecule to cell membranes and a precursor to bile acids and steroid hormones. Generally grouped into two categories, lipophilic and hydrophilic, statins differ structurally with polar or nonpolar moieties on their side chains. Hydrophilic statins (pravastatin and rosuvastatin) are absorbed more favorably within hepatic tissue compared to lipophilic statins (simvastatin, fluvastatin, lovastatin, pitavastatin, and atorvastatin) [3,9]. Interestingly, epidemiologic studies demonstrate a favorable antineoplastic profile for lipophilic statins over hydrophilic statins [10-12].

Statins inhibit cholesterol synthesis in the liver, resulting in decreased hepatic cholesterol concentration. As a result, hepatocyte LDL receptor expression increases, allowing circulating LDL molecules to move from the blood into the liver. Decreased LDL blood concentrations are associated with less blood vessel plaque buildup and limit the likelihood of atherosclerotic events, CVD, heart attack, and stroke. Statins also decrease blood triglyceride concentration, another indicator of CVD-related events [6,13,14].

Statins as anti-cancer therapies

The effects of statins are not limited to cholesterol inhibition. Numerous studies have investigated the pleiotropic properties of statins, one of which is anti-tumorigenesis. Statins exert anti-tumor effects on multiple hallmarks of cancer in different cancer types (breast, lung, bladder, prostate, etc.). HMGCR is a key enzyme in carcinogenesis and its inhibition appears to disrupt cancer pathogenesis [15]. Although preclinical studies seem to reach a consensus on the anti-tumor effects of statins, clinical study results have not garnered consistent, definitive conclusions on the benefit of statins in cancer patients. Here, we provide a brief background on both preclinical (namely cell culture and mouse model studies) and clinical literature regarding statins as anti-cancer treatments.

Various cancer cell lines are susceptible to statins because of their growth suppression and pro-apoptotic properties. Spampanato et al. note the induction of apoptosis via downregulation of Bcl-2 and upregulation of Bax in five different cancer cell lines after simvastatin administration [16]. Bcl-2 is an anti-apoptotic molecule that inhibits Bax, a mitochondrial channel protein. Bax allows pro-apoptotic molecules to induce the apoptotic caspase cascade. Additional studies have shown the same [17-19]. However, other studies have not been able to support the apoptotic effects of statins [20,21]. Statins also exert growth suppression on cancer cells. By interfering with the cell cycle, statins have been shown to reduce important cell cycle regulator molecules such as cyclin-dependent kinases (CDK) and their substrate partner, cyclins. Wang et al. describe the propensity of simvastatin to induce a G0/G1 cell cycle arrest in addition to the downregulation of CDK4/6 and Cyclin D1 [20]. In a study comparing various statins in human pancreatic cancer cell lines, Gbelcova et al. found that all statins except pravastatin inhibited K-Ras translocation to the cell membrane [22]. K-Ras mutations occur in over 90% of pancreatic cancers resulting in its permanent activation. K-Ras activation is associated with sustained growth in cancer. Statins also play a role in suppressing angiogenesis, invasion, and metastasis in preclinical studies [6,23-26].

Statin use is widespread and there are patients with cancer who have been on chronic long-term statin therapy. As such, retrospective cohort studies and meta-analyses have been conducted to show how the preclinical findings of statins and cancer fare from an epidemiological perspective. Four common outcomes in such studies are cancer incidence, mortality, recurrence, and survival. Outcomes vary greatly among these four measures. According to a review authored by Wang et al., two large studies looking at the relationship between statin use and all-cancer mortality concurred that statins were associated with a significantly lower risk of death and no significant reduction of cancer incidence [27]. The above applies to cancer broadly, but looking specifically at breast cancer, Beckwitt et al. acknowledge the reduction of breast cancer-specific mortality and cancer recurrence in a multitude of studies [6]. No significant relationship was found between statin use and breast cancer incidence in any of the studies cited by Beckwitt or Wang [6,27].

**Figure 1:**
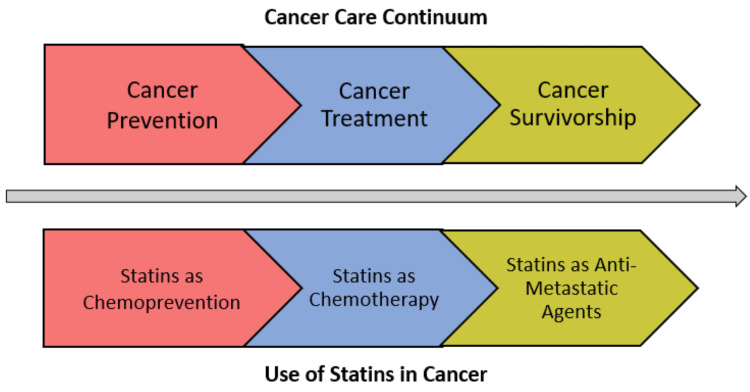
Cancer care continuum

Though it may appear there are protective effects of statins on breast cancer mortality and recurrence in the existing literature, further investigation should be completed to reveal the intricacies of the breast cancer population. Breast cancer is broadly grouped in many of the epidemiologic studies to date; however, breast cancer contains different molecular subtypes that respond differently to certain therapies. Additional factors, such as breast cancer subtype, stage, duration of treatment, type and dose of statin, and timing of treatment, are some factors that should be considered when studying the effects of statins on breast cancer. Clinical trials are needed to derive more meaningful conclusions about the efficacy of statins in breast cancer. To simplify the current data in a manner that connects the strides in animal models to the clinically relevant phases of cancer prevention and control, we have organized this systematic review according to the cancer continuum. We are not aware of any prior systematic reviews on the effects of statins in breast cancer animal models, and certainly not with the cancer care continuum framework (Figure 1). With this framework, we hope to connect lab science to the clinical context as a guide for clinicians and scientists.

Serving as the first category along the cancer care continuum, cancer prevention is defined as actions, strategies, or therapies implemented to reduce the risk of a cancer diagnosis. Maintaining a healthy lifestyle, avoiding exposure to known carcinogens, and chemoprevention are examples of cancer prevention tactics. In the case prevention is futile, accurate diagnosis and treatment of cancer are the next steps along the cancer care continuum patients will follow. Though the labels seen in Figure 1 are pictured as distinct sections of the cancer continuum, it is important to acknowledge some interventions do not fall into one single category. This framework serves as a useful tool in organizing the natural progression of cancer barring improvement. Treatment of cancer looks different for every patient; however, primary therapy is typically combined with neoadjuvant and/or adjuvant therapy to increase the likelihood of a cure and decrease the risk of recurrence. Finally, cancer survivorship encapsulates patients living with cancer in remission and striving to avoid relapse. When cancer has advanced to the metastatic stage, efforts shift from cure to options that slow the growth of metastases, extend life, or relieve symptoms. Statins have shown potential benefit in all three categories of the cancer care continuum in breast cancer and the present review summarizes related animal model findings.

## Review

Methods

The Preferred Reporting Items for Systematic Reviews and Meta-analyses (PRISMA) statement was followed for the systematic review [28]. To systematically review studies related to statins in breast cancer mouse models, an electronic search of the literature was conducted in the PubMed database. The following keywords were used, “mouse model” “statins,” and “breast cancer.” PubMed identified 58 articles, all of which were screened for potential eligibility. No studies were removed as duplicate records. Twenty-six studies met our bulleted inclusion and exclusion criteria and were included in our review.

Studies published as full-text articles, studies conducted in the past 15 years (2007-2022), breast cancer mouse models and the use of statin as exposure of interest were included. Non-full-text articles, cell culture and clinical studies, and studies exclusively on cancer types other than the breast were excluded.

Results

**Figure 2:**
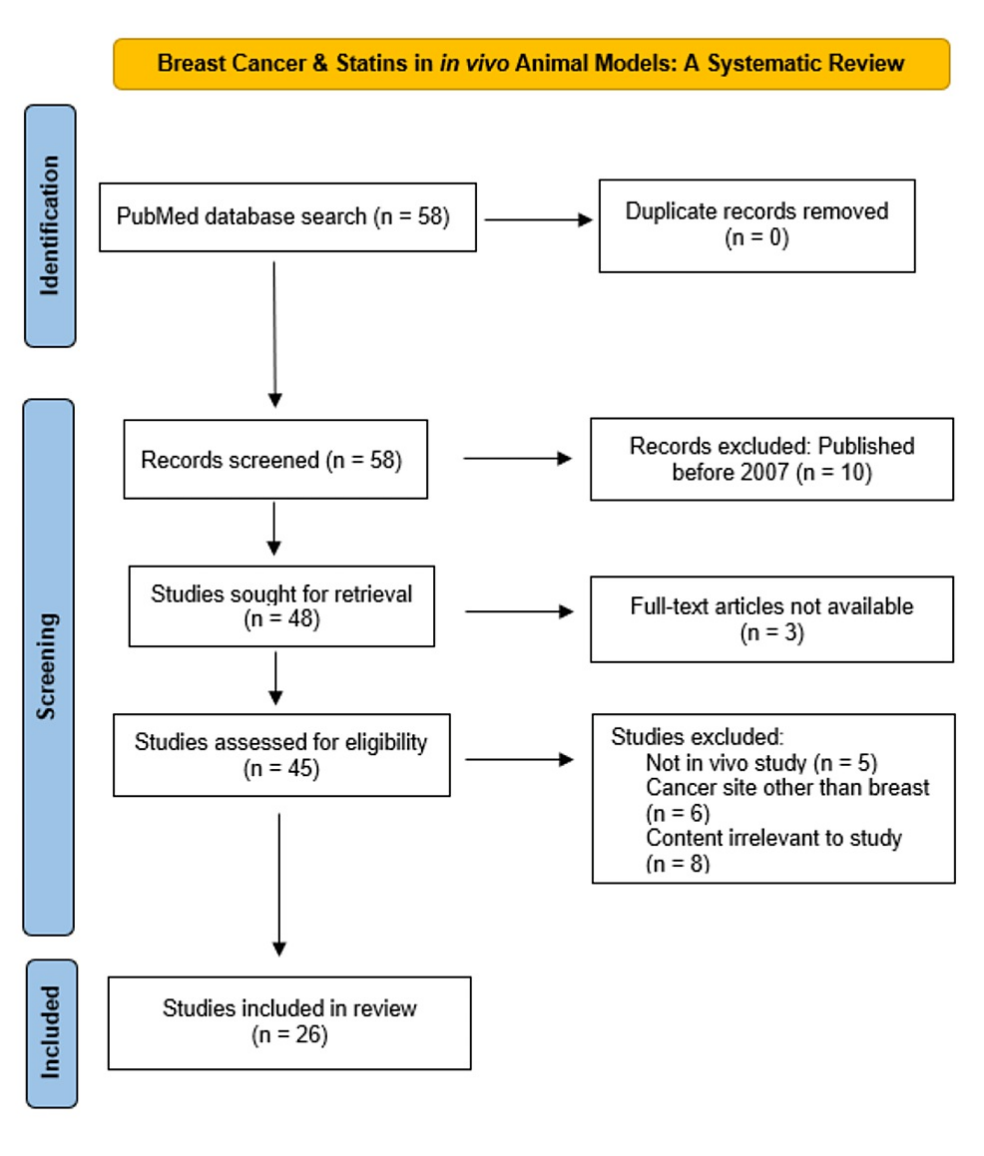
PRISMA flow diagram of studies identified in the systematic review

To keep our systematic review historically relevant, only articles from the past 15 years (January 1, 2007 to July 15, 2022) were to be included in the review. Of the 58 studies found in our initial search, 10 studies published prior to 2007 were excluded during initial screening, leaving 48 studies for additional eligibility screening. Full-text articles were a prerequisite to be included in our review, leaving three non-full-text articles excluded. Of the remaining 45 full-text articles, we screened their titles and abstracts for topical relevance to statins and breast cancer in animal models. Nineteen studies were excluded from the analysis: eight studies included content not relevant to the review, six studies investigated exclusively a cancer type other than breast, and five studies did not study an in vivo animal model. Of the articles remaining, 26 matched our criteria and were to be included in the review as shown in Figure 2. These 26 studies were placed in one of the three categories along the cancer care continuum according to Figure 1. Three studies covered statins as chemoprevention, 14 on statins as chemotherapy (five studies on the tumor-suppressive properties of statins alone and nine studies on statins as part of combination therapy), and nine studies on the anti-metastatic properties of statins in breast cancer.

Discussion

The summarized results from the 26 studies included in our systematic review can be found in Table 1. Overall, statins both alone and in combination were found to have dose-dependent antineoplastic effects. However, when combined with another anti-cancer agent, statins appeared to have a greater effect compared to statins alone, suggesting a synergistic benefit with established anti-cancer agents. The studies classified as chemoprevention found significantly delayed tumor onset, albeit using only one statin [29-31]. Statin monotherapy studies saw the downregulation of common biomarkers that are often mutated in breast cancer [32-36]. Combination therapy studies noted more dramatic decreases in tumor size, weight, and volume than monotherapy. Also, different sub-categories of combination therapy based on oncogenic molecular pathways or cancer subtypes emerged in our search. The Wnt signaling pathway, HER2-positive breast cancer, hormone receptor-positive breast cancer, and histone deacetylase inhibitor therapy produced their own unique findings [37-45]. Finally, antimetastatic studies, through the use of a variety of statins, significantly reduced metastases in the brain, bone, liver, and lung [23,46-53].

**Table 1:** Overview of included studies EtOH: ethanol; TNBC: triple-negative breast cancer; qPCR: quantitative polymerase chain reaction; HMGCR: 3-hydroxy-3-methylglutaryl-CoA reductase; BRCA1: breast cancer gene 1; BrdU: bromodeoxyuridine; CDK4: cyclin-dependent kinase 4; pRb: retinoblastoma protein; DMBA: 12-dimethylbenz(a)anthracene; DMSO: dimethyl sulfoxide; PTEN: phosphatase and tensin homolog; AKT: protein kinase B; RhoB: ras homolog family member B; Bcl: B-cell lymphoma 2; MCM7: minichromosome maintenance complex component 7; HDACi: histone deacetylase inhibitor; HER2: human epidermal growth factor receptor 2; Erbb2: Erb-B2 receptor tyrosine kinase 2; MNU: methylnitrosourea; ER: estrogen receptor; DKK-1: dickkopf-1; PSV: polyethylene glycol-s-s-vitamin E succinate; Ki-67: antigen KI-67; Gy: gray

Author (Year)	Study Design	Animal Model(s)	Outcomes Measured	Findings/Conclusions
Statins as Chemoprevention				
Bhardwaj (2021) [27]	Fluvastatin (10 mg/kg/day) vs. Vehicle control (0.001% EtOH) for 16 wks	5-6 wk old SV40C3 TAg transgenic TNBC mouse model	Tumor incidence, multiplicity, weight, latency	Significant delay in onset of tumors (20 wks vs. 16.8 wks) and 75% reduction in tumor weight compared to control.
Bhardwaj (2022) [28]	Fluvastatin (10 mg/kg/day) vs. Vehicle control (0.001% EtOH) for 16 wks	5-6 wk old SV40C3 TAg transgenic TNBC mouse model	Tumor histologic grade and qPCR evaluation of fluvastatin-resistant gene signatures	Upregulation of cholesterol biosynthesis pathway genes in mice mammary tumors is strongly associated with resistance to statin chemoprevention.
Bhardwaj (2018) [29]	Fluvastatin (10 mg/kg/day) vs. Control (water) for 16 wks	5 wk old MCF10.AT1 xenograft TNBC mouse model	Tumor size, histologic grade, HMGCR mRNA level	25% reduction in tumor size of fluvastatin-treated mice compared to control. No difference in histologic grade of tumors between groups.
Statins as Chemotherapy				
Monotherapy				
Karimi (2019) [30]	Simvastatin (40 or 80 mg/kg/day) vs. tamoxifen (50 mg/kg/day) vs. Control (sesame oil)	5 wk old DMBA-treated mouse model (50 mg/kg/day)	Tumor size, weight, volume, oxidative stress biomarkers of serum, mammary glands, and tumors	Tamoxifen and high dose simvastatin improved carcinogenic parameters and lessened the severity of oxidative stress, a driver of breast cancer progression, compared to control.
Ghosh Choudhury (2010) [31]	Simvastatin (5 mg/kg/day) vs. control (phosphate-buffer saline) for 1 wk	2 wk old MDA-MB-231 mammary tumoral xenograft mouse model	Quantification of phosphorylated AKT, Bcl, PTEN	Simvastatin significantly increases levels of tumor suppressor PTEN while simultaneously downregulating anti-apoptotic molecule Bcl.
Ma (2019) [32]	Atorvastatin (10 mg/kg/day) vs. DMSO control	4 wk old MCF-7 intramammary tumoral xenograft mouse model	Tumor size, volume, weight, mRNA/protein expression of RhoB, PTEN, AKT	Atorvastatin has carcinostatic effects that act via the PTEN/AKT pathway.
Li (2017) [33]	Simvastatin (60 mg/kg/day) vs. control (water) for 10 days	4T1 breast carcinoma xenograft mouse model	Tumor size, weight, volume, MCM7 and RB protein expression, %MCM7+ and RB+ cells	Decreased expression of RB and MCM7 create genome instability and thus induce apoptosis in simvastatin-treated mammary carcinoma mice xenografts.
Yu (2008) [34]	Lovastatin (10 mg/kg/day) vs. Placebo (0.9% NaCl) for 2 wks	8 wk old MCF-7/BRCA1 tumoral xenograft mouse model	Anti-proliferation via BrdU and mRNA/protein expression of cyclin D1, CDK4, pRb, and p21	BRCA1 overexpression revealed significantly enhanced anti-proliferation and reduced cell cycle mRNA and protein levels in lovastatin-treated breast tumoral xenograft mice models.
Combination Therapy				
Wnt Signaling Pathway				
Sulaiman (2018) [35]	Simvastatin + Wnt inhibitor ICG-001 & atorvastatin + zoledronic acid, respectively	MDA-MB-231 xenograft TNBC mouse model	Tumor weight, relative cancer stem cell concentrations, survival, serum DKK-1 levels	Statin and Wnt inhibitor combination therapy shows reduction in tumor weight and cancer stem cell populations for both epithelial and mesenchymal phenotypes. No concrete conclusions can be drawn from decreased DKK-1 levels.
Göbel (2015) [36]				
HER2+ breast cancer				
Oechsle (2020) [37]	Simvastatin + dendritic cell-based immunotherapy & lovastatin + lapatinib, respectively	HCC1954 & TUBO xenograft HER2+ mouse models, respectively	Tumor size, volume, weight, relative Erbb2 protein%	Two different combination therapy regimens showed synergistic benefit of overall tumor reduction in HER2+ breast cancer animal models. Molecular concentrations of Erbb2 protein are reduced as well.
Zhang (2019) [38]				
HR+ breast cancer				
Liang (2017) [39]	Simvastatin + tamoxifen & atorvastatin + tamoxifen, respectively	Tamoxifen-resistant MCF7 xenograft mouse model & MNU-induced ER+ rat model, respectively	Tumor size, weight, volume, incidence, multiplicity, MCM7 protein expression	In optimal doses, statin plus tamoxifen is synergistic against hormone receptor positive tumors. However, in suboptimal doses, limited synergistic potential exists.
Lubet (2009) [40]				
HDACi therapy				
Kou (2017) [41]	Simvastatin + vorinostat & mevastatin + pabinostat, respectively	MDA-MB-231 xenograft TNBC mouse model for all studies	Tumor size, volume, weight, survival, apoptosis biomarkers	Combination HDACi therapy with statins induced significant growth suppression and apoptosis compared to monotherapy or control. Future preclinical and clinical studies should seek to investigate the effect of chemotherapy and other statins.
Kou (2018) [42]				
Lin (2017) [43]				
Statins as Anti-metastatic agents				
Beckwitt (2018) [23]	Atorvastatin (2 mg/kg/day) vs. atorvastatin (10 mg/kg/day) vs. vehicle control for 3 wks	8 wk old NSG MDA-MB-231 liver & lung metastatic mouse model	Tumor size and proliferation, metastatic burden and proliferation in lung and liver models	Atorvastatin shows dose-dependent decreases in metastatic proliferation, but not primary tumor cell proliferation in both lung and liver metastasis models.
Xu (2014) [44]	Atorvastatin-loaded PSV micelle (5 mg/kg/day) vs. free atorvastatin (5 mg/kg/day) vs. control (saline) for 18 days	4T1 orthotopic mammary tumor metastatic cancer mouse model	Animal weight, tumor volume, number of metastatic nodules	Atorvastatin-loaded PSV micelles significantly reduced the number of pulmonary metastatic nodules by 84.8% compared to free atorvastatin.
Wolfe (2015) [45]	Simvastatin (15 mg/kg/day) vs. control (DMSO) for 7 wks	4 wk old SUM 149 & MDA-MB-231 orthotopic & tail vein injection metastasis mouse model	Number of lung and brain metastases, metastasis free survival	Significantly lower number of brain metastases and longer metastasis free survival is seen in simvastatin treated mice.
Howe (2020) [46]	Pitavastatin (1 mg/kg/day) vs. simvastatin (5 mg/kg/day) vs. vehicle control	MDA-231 intracardiac & intracranial breast cancer brain metastasis mouse model	Brain mets incidence, Ki-67 staining quantification, survival	Decreased brain metastasis incidence, decreased metastatic proliferation, and increased survival are apparent in statin treated metastasis mouse models.
Mandal (2011) [47]	Simvastatin (5 mg/kg/day) vs. control (phosphate-buffer saline) for 1 wk	MDA-MB-231 mammary tumor bone metastasis mouse model	Osteolytic lesion visualization and quantification	Compared to control mice, simvastatin-treated mice demonstrated significantly reduced bone lesions and thus prevents breast cancer bone metastasis.
Wang (2019) [48]	Pitavastatin (4 or 8 mg/kg/day) vs. vehicle control for 3 wks	6 wk old 4T1.2 tibial bone metastasis mouse model	Tibial visualization, bone volume, bone mineral density, number of osteolytic lesions	In an osteolytic model of breast cancer, high dose pitavastatin played a protective role in bone metastasis compared to placebo.
Vintonenko (2012) [49]	Fluvastatin (15 mg/kg/day) vs. zoledronate (100 µg/kg) vs. control (phosphate-buffered saline) for 3 wks	Intracardiac bioluminescent MDA-MB-231 TNBC mouse model	Bioluminescent signal of treated mice, number of detected metastatic sites, survival of treated mice	Fluvastatin treatment reduced the overall metastatic burden compared to control and contributed increased survival compared to the other 2 groups.
Marti (2021) [50]	Atorvastatin (10 mg/kg/day) + doxorubicin (2 mg/kg)/paclitaxel (10 mg/kg)	MDA-MB-231 spleen to liver xenograft	Metastatic proliferation quantification	Doxorubicin, but not paclitaxel, combined with statins show potential useful benefit in a primary spleen to metastatic liver mouse model.
Efimova (2018) [51]	Pitavastatin (20 mg/kg/day) + 6Gy tumor irradiation	MCF7 xenograft mouse model	Tissue damage & cellular senescence	Alone, pitavastatin has minimal effects on breast tumor-bearing mice, however, in combination with ionizing radiation, DNA damage and decreased proliferation are enhanced.

Statins As Chemoprevention

The three studies investigating statin chemoprevention in this review exclusively modeled the progression of triple-negative breast cancer (TNBC), an aggressive form of breast cancer histologically known for absent to low concentrations of estrogen (ER), progesterone (PR), and human epidermal growth factor 2 (HER2) protein receptors. As the molecular breast cancer phenotype with the most aggressive histology and poorest outcomes, it tends to be more commonly studied in the context of statins in an effort to reveal the efficacy and mechanisms of novel and repurposed therapies [29-31].

Both transgenic mice models and breast cancer mice xenografts have been studied in statin chemoprevention. In a study by Bhardwaj, transgenic mice were treated with fluvastatin. Tumor incidence and growth were reduced by the end of the treatment window. Because it was known that invasive cancer typically presented at the age of 16 weeks in this transgenic model, fluvastatin therapy was administered in five to six-week-old mice to adequately study statin chemoprevention. Although the number of mice that developed tumors as well as the number of tumors per mouse was reduced in fluvastatin-treated mice, the data did not reach statistical significance when compared to the control. Tumor weight in fluvastatin-treated mice was significantly reduced compared to the control, providing evidence for the tumor-suppressive properties of statins. Additional mechanistic experiments concluded that increased apoptosis was responsible for the reduced tumor incidence findings [29]. In a second study conducted by the same author, Bhardwaj et al. used identical transgenic mice models to look at gene clusters associated with fluvastatin resistance. They hypothesized that certain gene signatures were connected to the progression of preneoplastic lesions. They ultimately concluded that the upregulation of certain cholesterol biosynthesis genes such as HMGCR could contribute to driving the invasive disease. The authors highlighted the importance of screening and identifying women with associated gene upregulations and suggest avoiding statin therapy for these subsets due to their resistant phenotype [30]. The authors also recommended targeting statin therapy to at-risk populations more likely to respond. These efforts will hopefully allow for the optimization of statins as chemopreventative agents in breast cancer.

Mice xenografts were also studied in the context of statin chemoprevention in a third study by Bhardwaj et al. After injecting a well-established TNBC cell line into female mice, fluvastatin was started one week later. Compared to control mice, lesions treated with fluvastatin were 25% smaller, however, post-sacrifice, the histology of the fluvastatin-treated and control-treated xenografts did not differ under the microscope. No difference in tumor grade led the authors to conclude statin therapy did not slow the progression of tumors [31]. Conflicting results in statin chemoprevention animal studies demonstrate the need for additional research to generate a diverse study pool with clearer conclusions. Future areas of research could include studying different breast cancer xenograft and transgenic mouse models to illustrate how efficacious statins truly are in cancer prevention. Just as previously mentioned, TNBC was the focus of chemoprevention studies thus opening the opportunity for hormone-positive breast cancer animal models to be studied in the future. In the same way, the efficacy of other statins should be considered (hydrophilic vs. lipophilic) to reveal any benefits or pitfalls each statin may exhibit.

Statins As Chemotherapy

Monotherapy: Across the five studies investigating the chemotherapeutic effect of statins as monotherapy, administration of statin therapy significantly reduced the size, weight, and volume of breast cancer tumors compared to control mice whether injected to create xenograft or induced by chemical carcinogen [32-36]. Typical tumor parameters such as tumor size, weight, and volume were measured in all studies, however, each study measured additional cancer biomarkers to track the molecular mechanisms of statin tumor suppression. Karimi et al. looked at biomarkers associated with oxidative stress to find the administration of simvastatin at a higher dose lessened the severity of oxidative stress, a consistent phenotype that fuels breast cancer growth [32]. Two studies using different breast cancer cell xenografts demonstrated significantly increased PTEN expression in simvastatin and atorvastatin-treated mice respectively [33,34]. PTEN acts as a tumor suppressor in the AKT cell survival pathway. By use of phosphatase activity, PTEN reverses the action of the kinase PI3K to effectively prevent cell proliferation and inhibition of apoptosis. In a study conducted by Li et al., simvastatin-treated mice showed significantly decreased expression of tumor suppressor RB and DNA replication licensing factor MCM7 in addition to increased PTEN expression. Mechanistically, lowered quantities of RB and MCM7 resulted in chromosome instability and thus induction of apoptosis [35]. Finally, Yu et al. found that lovastatin-treated mice with overexpression of the tumor suppressor BRCA1 revealed reduced proliferation when compared to tumor xenografts expressing normal BRCA1 amounts. mRNA and protein expression of cell cycle progressor molecules cyclin D1 and CDK4 were significantly reduced in lovastatin-treated mice xenografts when compared to placebo-treated mice. Similarly, lovastatin-treated mice with BRCA1 overexpression had significantly lowered cyclin D1 and CDK4 compared to the mice with regular expression of BRCA1 even though both mice xenografts were given lovastatin. The authors concluded that BRCA1 overexpression sensitized breast tumoral xenograft models to lovastatin [36].

In summary, the tumor suppressive effects of statins widely target many different cell proliferative and apoptotic pathways in breast cancer. Identification of molecular targets such as MCM7 and BRCA1 is important in elucidating the detailed mechanisms of statin therapy in breast cancer. Clinically, these cancer biomarkers could possibly serve as tools in identifying subpopulations with breast cancer that could benefit the most from statin therapy. As next-generation sequencing is becoming less expensive, this possibility is within sight. There is no clinical likelihood of prescribing a statin as exclusive primary therapy to treat breast cancer, but these single-agent studies introduce the contributory and intrinsic antineoplastic value of statins. The application of this information in human breast cancer might be the pharmacogenetic screening of pertinent genes and proteins.

Combination therapy: There were nine articles that examined statins as combination therapy in the treatment of animals modeling breast cancer [37-45]. Four key areas were common to statin combination therapy studies: Wnt-signaling pathways, HER2-positive breast cancer, hormone receptor-positive breast cancer, and histone deacetylase inhibitor therapy (HDACi).

The Wnt/β-catenin pathway provides tumor-driving signals to cancer cells and contributes to the overall pathogenicity of cancer. Pertinent to the morphologic transition of cancer cells from epithelial to mesenchymal (as well as vice versa), Wnt and its associated downstream signaling partners drive tumor growth and metastasis. A study conducted by Sulaiman et al. observed a positive synergistic effect between the Wnt inhibitor ICG-001 and simvastatin in a human xenograft model of TNBC. Dual administration of ICG-001 and simvastatin significantly reduced tumor weight and associated cancer stem cells (CSCs) in both mesenchymal and epithelial phenotypes compared to a single drug and vehicle treatment. CSCs represent a small subpopulation of tumor cells with self-renewal and differentiation potential, making them important targets for anti-cancer therapy. Interestingly, epithelial and mesenchymal tumors treated with Wnt/statin combination therapy displayed decreased tumor initiation after secondary transplantation into new mice when compared to control and single drug-treated mice [37]. In another study relevant to the Wnt pathway, researchers identified how suppression of the Wnt inhibitor Dickkopf-1 (DKK-1) by intratumoral administration of atorvastatin and zoledronic acid could potentially limit osteolytic bone lesions as a complication of malignant breast cancer. Though a significant portion of the article explores the effects of combination therapy in various cancer cell lines, serum DKK-1 concentrations in xenografted mice were reduced by 25% in treated mice compared to untreated [38]. Although the authors did show reduced DKK-1 concentrations in combination-treated mice with breast cancer, parameters to show the global suppression of tumors, such as tumor weight and volume, were not recorded. Additionally, no biomarkers showing potential therapeutic effects on metastatic bone lesions were measured. The clinical relevance of the downregulation of tumor DKK-1 is yet unknown, but additional in vivo testing might elucidate the possible link to breast cancer bone metastases.

Statin combination therapies have been studied in relation to HER2-positive (also known as Erbb2-positive) breast cancer. HER2 is an oncogenic driver of breast cancer but is susceptible to targeted therapies, many of which harness the immune system to halt sustained growth and cell survival pathways. In a HER2-positive breast cancer mouse model, concurrent treatment of simvastatin and a dendritic cell (DC)-based immunotherapy showed approximately 60% smaller tumors when compared to single treatments and control. Simvastatin treatment was given five times a week for three weeks, while the DC-based immunotherapy was administered twice weekly for the same three weeks. In an additional experiment from the same article, HER2-positive mice were treated with both simvastatin and recombinant interferon-gamma (IFN-γ), a cytokine thought to mediate the effects of the previous DC-based immunotherapy. Similar results revealed that IFN-γ plus simvastatin significantly reduces tumor size as well. Oechsle et al. concluded that there is a synergistic benefit simvastatin provides for DC-based immunotherapy in HER2-positive mice [39]. Although the immune system can be used to disrupt the growth of HER2-positive breast cancer, inhibition of the HER2 ATP-binding domain in conjunction with statins appears to have similar effects. The Erbb2 inhibitor lapatinib, when combined with lovastatin, significantly decreased tumor volume and weight compared to control-treated and lapatinib-treated HER2-positive xenografted mice. Relative Erbb2 was also reduced in mice that received combination statin plus lapatinib compared to lapatinib monotherapy. This study provides additional mechanistic evidence contending that lower membrane cholesterol, an effect mediated by statins, contributes to Erbb2 receptor endocytosis and eventual degradation. This degradation allows drugs such as lapatinib to suppress HER2-positive breast cancer more effectively [40].

Hormone receptor-positive breast cancer comprises another subset of breast cancer important to understand when discussing treatment. Typically, patients with breast cancers identified as hormone receptor-positive (ER+ and/or PR+) have better prognoses and longer survival. Drugs that work to inhibit ER and PR receptors and their downstream growth signaling pathways (known as endocrine therapy) are customary first-line therapies in hormone receptor-positive breast cancer. However, breast cancer cells can sometimes become resistant to endocrine therapy leaving patients susceptible to further progression of their cancer. Statins may provide some relief for endocrine therapy-resistant breast cancer according to a study conducted by Liang et al. When simvastatin and tamoxifen, a selective estrogen receptor modulator, are combined in tamoxifen-resistant xenografted mice models, tumor size, weight, and volume were markedly reduced compared to placebo mice. Moreover, immunohistochemical staining revealed lower MCM7 expression similar to the study by Li with simvastatin as monotherapy [35,41]. The data from Liang support that simvastatin and tamoxifen combination therapy results in the downregulation of MCM7 and thus induction of apoptosis. Lubet et al., in a methylnitrosurea-induced ER-positive rat model, showed that suboptimal doses of tamoxifen, when combined with atorvastatin, did not significantly impact tumor incidence or multiplicity when compared to the control. Similar results were demonstrated when rats were administered atorvastatin and bexarotene, a retinoid X receptor agonist. Additional experiments revealed lovastatin and bexarotene show limited synergistic potential in this model of breast cancer [42]. Although hormone-positive breast cancer has a more favorable prognosis with good existing treatment options, there could be a potential benefit of statins in the scenario of endocrine-resistant cancers.

HDACi have emerged as effective anticancer drugs that play a role in regulating gene expression. Their potential synergistic benefit with statins for the treatment of TNBC has been investigated. Xenografted mice were treated with simvastatin, vorinostat, a common HDACi, or both. Like most other studies, combined treatment significantly reduced tumor size and volume without any major toxicity. Researchers also performed an assay to visualize upregulated apoptosis in combination therapy and quantified the significantly increased levels of apoptosis. By increasing the expression of tumor-suppressor genes, vorinostat’s ability to induce apoptosis is thought to be enhanced by simvastatin [43]. In two similar studies, another HDACi labeled LBH589 plus statin was studied in TNBC xenografted mice models. These two studies looked at different statins, one simvastatin, and the other mevastatin. Both studies produced similar results, displaying pronounced growth suppression in mice receiving combination therapy compared to mice receiving monotherapy or control [44,45]. HDACi therapy has shown partial efficacy in clinical trials, however, the most benefit is seen when combined with traditional chemotherapeutic agents. Given the promising results from the mentioned animal studies, statins could potentially become part of the standard treatment regimen of HDACi therapy, although clinical trials should assess this first. HDACi-eligible patients already on statins for heart and vascular health are likely not at any increased risk of morbidity or mortality given the proven safety profile of statins. However, HDACi-eligible patients at risk for potential drug-drug interactions should be adequately screened before prescribing.

Combination therapy with statins and either standard or experimental therapeutics has proven beneficial in breast cancer animal models. Synergistic benefits are not exclusive to one subtype of breast cancer as TNBC, HER2-positive, and hormone-positive breast cancer are all sensitive and susceptible phenotypes to statin combinatory therapy in vivo. Questions remain about the dose, type, and timing of statin therapy, and future directions of animal studies that could address these parameters.

Statins As Anti-metastatic Agents

Metastatic breast cancer is typically treated with palliative rather than curative intent, as cytotoxic therapies can be used to slow the growth of the disease or alleviate associated symptoms. Animal model evidence portrays statins as a possible nontoxic agent capable of slowing breast cancer metastasis at common sites including the lungs, liver, brain, and bones. In separate breast cancer metastasis mouse models, Beckwitt et al. measured liver and lung metastatic proliferation in atorvastatin-treated mice. Liver size did not significantly vary, however, under the microscope, the proliferation of metastatic cells decreased and varied dose-dependently in atorvastatin-treated mice. Almost identical results were found in the lung metastasis mouse model, but no statistical significance was achieved in the dose-dependent decreases in metastatic proliferation [25]. Typically, statins are administered to mice via injection or diet, but a novel nano-drug delivery system created by Xu et al. called atorvastatin-loaded PSV micelles was tested to suppress metastasis of breast cancer mice models. The number of metastatic pulmonary nodules was significantly reduced by 84.8% compared to atorvastatin delivery without PSV micelles. This promising novel drug delivery mechanism warrants the study of combination therapies using PSV micelles [46]. Distant brain metastases are also affected by simvastatin pretreatment of breast cancer mouse models. Whether SUM 149 cancer cells were injected orthotopically or via tail vein, DMSO control mice developed more brain Mets and had shorter metastasis-free survival than the simvastatin-treated group in a study conducted by Wolfe et al. These results were statistically significant [47]. Another study corroborates the evidence for decreased brain metastasis in both intracardiac and intracranial models of breast cancer [48]. Bones are another structure commonly affected by breast cancer metastasis. One study provides x-ray evidence of markedly reduced osteolytic lesions in simvastatin-treated mice compared to control, thus demonstrating the potential for prevention of distant bone metastasis in breast cancer [49]. Pitavastatin also was shown to exhibit similar anti-metastatic properties in osteolytic lesions of mice tibias [50]. Finally, three weeks of fluvastatin treatment reduced the overall metastatic burden of a xenograft mouse model [51]. With both global and organ-specific benefits on metastatic breast cancer animal models, statins should be further studied in prospective trials to enhance the existing body of knowledge on the clinical relevance of their anti-metastatic properties.

Lastly, statins have been used to sensitize cancer cells to common chemotherapeutic agents, namely doxorubicin, as well as ionizing radiation in metastatic mice models. Marti et al. primarily studied the effects of combinatory atorvastatin and chemotherapy on TNBC. In a primary spleen-to-liver metastasis model of breast cancer, cells were injected into mice spleens to establish an ectopic primary tumor. Mice were pretreated with atorvastatin for three weeks and subsequently received concurrent treatment with either doxorubicin or paclitaxel. The addition of atorvastatin did not improve the efficacy of either chemotherapy drug when looking at the primary splenic tumor. However, atorvastatin did slow the metastasis of cancer cells to the liver when combined with doxorubicin, but not paclitaxel. The fraction of cells entering the S-phase of the cell cycle visualized by EdU+ uptake was significantly reduced in atorvastatin plus doxorubicin-treated mice when compared to doxorubicin-treated mice. Atorvastatin plus paclitaxel-treated mice did not demonstrate the same results [52]. The authors underscore the powerful cytocidal effects statins provide in preclinical models, which illustrates the need for additional randomized clinical studies to clarify how efficacious statins can be when combined with chemotherapy against metastatic TNBC. Another common treatment option, when faced with a cancer diagnosis, is radiation therapy. In a study conducted by Efimova et al., mice injected with a tumor were administered pitavastatin followed by 6 Gy of tumor irradiation. Co-treatment of pitavastatin with radiation-induced tissue damage and cellular senescence in a similar way to the PARP inhibitor veliparib [53]. These results convey the need for clinical studies to confirm the potentiating effect of statins on radiotherapy for the treatment of metastatic breast cancer.

Limitations

There are limitations. Mouse studies entail a shorter course of disease development and statin exposure. Additionally, mouse models do not reliably predict human toxicity or the efficacy of oncologic interventions, making them imperfect replicas to study breast cancer and statin interventions. Finally, the creation of cancer mouse models relies on the injection of preexisting cancer cell lines, which misaligns with how cancer can typically emerge in humans (carcinogen exposure, genetic instability, and heritable mutations). Our study also has its limitations. We cannot rule out the possibility of publication bias in the studies included in our systematic review. Positive results tend to be published more than negative results and there is a risk of publication bias in our study.

## Conclusions

This systematic review summarizes existing preclinical mouse studies investigating the benefits statins provide in breast cancer. We adopted a cancer care continuum framework to provide a clinically relevant picture for clinicians and scientists alike. As demonstrated, statins exhibit a range of anti-cancer effects in preclinical breast cancer animal models that reach across the cancer care continuum. Breast cancer mouse models have provided important experimentation for statins and combination therapy. Mouse models tend to be the most common animal model in studies examining the effect of statins on breast cancer. Although mouse models have provided evidence of statins as monotherapy, this would never be clinically appropriate, but the potential benefit to add statins as part of combination therapy could serve useful for clinical practice after being thoroughly vetted in clinical studies. We emphasize and caution against translating the results of animal studies into clinical practice. Further clinical studies would need to be required to support statin use as part of a breast cancer treatment regimen. Moreover, just as mouse studies attempt to reveal findings on molecular subtypes of breast cancer, clinical studies should attempt to do the same, and it seems that this area of clinical research is starting to emerge. The results of these studies at both the preclinical and clinical levels are key in determining that there may be a role for statins in cancer prevention or treatment algorithms for patients diagnosed with breast cancer or as agents to improve survivorship.

## References

[1] Endo A (2010). A historical perspective on the discovery of statins. Proc Jpn Acad Ser B Phys Biol Sci.

[2] Hajar R (2011). Statins: past and present. Heart Views.

[3] Fong CW (2014). Statins in therapy: understanding their hydrophilicity, lipophilicity, binding to 3-hydroxy-3-methylglutaryl-CoA reductase, ability to cross the blood brain barrier and metabolic stability based on electrostatic molecular orbital studies. Eur J Med Chem.

[4] Sirtori CR (2014). The pharmacology of statins. Pharmacol Res.

[5] Ward NC, Watts GF, Eckel RH (2019). Statin toxicity. Circ Res.

[6] Beckwitt CH, Brufsky A, Oltvai ZN, Wells A (2018). Statin drugs to reduce breast cancer recurrence and mortality. Breast Cancer Res.

[7] Buhaescu I, Izzedine H (2007). Mevalonate pathway: a review of clinical and therapeutical implications. Clin Biochem.

[8] Istvan ES, Deisenhofer J (2001). Structural mechanism for statin inhibition of HMG-CoA reductase. Science.

[9] Climent E, Benaiges D, Pedro-Botet J (2021). Hydrophilic or lipophilic statins?. Front Cardiovasc Med.

[10] Ahern TP, Lash TL, Damkier P, Christiansen PM, Cronin-Fenton DP (2014). Statins and breast cancer prognosis: evidence and opportunities. Lancet Oncol.

[11] Ahern TP, Pedersen L, Tarp M (2011). Statin prescriptions and breast cancer recurrence risk: a Danish nationwide prospective cohort study. J Natl Cancer Inst.

[12] Desai P, Lehman A, Chlebowski RT (2015). Statins and breast cancer stage and mortality in the Women's Health Initiative. Cancer Causes Control.

[13] Stancu C, Sima A (2001). Statins: mechanism of action and effects. J Cell Mol Med.

[14] Smith MEB, Lee NJ, Haney E, Carson S (2009). Drug Class Review: HMG-CoA reductase inhibitors (Statins) and fixed-dose combination products containing a statin: final report update 5. Portland.

[15] Sławińska A, Kandefer-Szerszeń M (2008). [The anticancer properties of statins]. Postepy Hig Med Dosw (Online).

[16] Spampanato C, De Maria S, Sarnataro M (2012). Simvastatin inhibits cancer cell growth by inducing apoptosis correlated to activation of Bax and down-regulation of BCL-2 gene expression. Int J Oncol.

[17] Goc A, Kochuparambil ST, Al-Husein B, Al-Azayzih A, Mohammad S, Somanath PR (2012). Simultaneous modulation of the intrinsic and extrinsic pathways by simvastatin in mediating prostate cancer cell apoptosis. BMC Cancer.

[18] Buranrat B, Suwannaloet W, Naowaboot J (2017). Simvastatin potentiates doxorubicin activity against MCF-7 breast cancer cells. Oncol Lett.

[19] Kotamraju S, Williams CL, Kalyanaraman B (2007). Statin-induced breast cancer cell death: role of inducible nitric oxide and arginase-dependent pathways. Cancer Res.

[20] Wang G, Cao R, Wang Y (2016). Simvastatin induces cell cycle arrest and inhibits proliferation of bladder cancer cells via PPARγ signalling pathway. Sci Rep.

[21] Zhao XH, Xu ZR, Zhang Q, Yang YM (2012). Simvastatin protects human osteosarcoma cells from oxidative stress-induced apoptosis through mitochondrial-mediated signaling. Mol Med Rep.

[22] Gbelcová H, Lenícek M, Zelenka J (2008). Differences in antitumor effects of various statins on human pancreatic cancer. Int J Cancer.

[23] Weis M, Heeschen C, Glassford AJ, Cooke JP (2002). Statins have biphasic effects on angiogenesis. Circulation.

[24] Park HJ, Kong D, Iruela-Arispe L, Begley U, Tang D, Galper JB (2002). 3-hydroxy-3-methylglutaryl coenzyme A reductase inhibitors interfere with angiogenesis by inhibiting the geranylgeranylation of RhoA. Circ Res.

[25] Beckwitt CH, Clark AM, Ma B, Whaley D, Oltvai ZN, Wells A (2018). Statins attenuate outgrowth of breast cancer metastases. Br J Cancer.

[26] Denoyelle C, Vasse M, Körner M (2001). Cerivastatin, an inhibitor of HMG-CoA reductase, inhibits the signaling pathways involved in the invasiveness and metastatic properties of highly invasive breast cancer cell lines: an in vitro study. Carcinogenesis.

[27] Wang A, Wakelee HA, Aragaki AK, Tang JY, Kurian AW, Manson JE, Stefanick ML (2016). Protective effects of statins in cancer: should they be prescribed for high-risk patients?. Curr Atheroscler Rep.

[28] Moher D, Liberati A, Tetzlaff J, Altman DG (2009). Preferred reporting items for systematic reviews and meta-analyses: the PRISMA statement. PLoS Med.

[29] Bhardwaj A, Embury MD, Rojo RD, Albarracin C, Bedrosian I (2021). Efficacy of fluvastatin and aspirin for prevention of hormonally insensitive breast cancer. Breast Cancer Res Treat.

[30] Bhardwaj A, Embury MD, Ju Z, Wang J, Bedrosian I (2022). Gene signature associated with resistance to fluvastatin chemoprevention for breast cancer. BMC Cancer.

[31] Bhardwaj A, Singh H, Trinidad CM, Albarracin CT, Hunt KK, Bedrosian I (2018). The isomiR-140-3p-regulated mevalonic acid pathway as a potential target for prevention of triple negative breast cancer. Breast Cancer Res.

[32] Karimi B, Ashrafi M, Shomali T, Yektaseresht A (2019). Therapeutic effect of simvastatin on DMBA-induced breast cancer in mice. Fundam Clin Pharmacol.

[33] Ghosh-Choudhury N, Mandal CC, Ghosh-Choudhury N, Ghosh Choudhury G (2010). Simvastatin induces derepression of PTEN expression via NFkappaB to inhibit breast cancer cell growth. Cell Signal.

[34] Ma Q, Gao Y, Xu P (2019). Atorvastatin inhibits breast cancer cells by downregulating PTEN/AKT pathway via promoting Ras homolog family member B (RHOB). Biomed Res Int.

[35] Li J, Liu J, Liang Z (2017). Simvastatin and atorvastatin inhibit DNA replication licensing factor MCM7 and effectively suppress RB-deficient tumors growth. Cell Death Dis.

[36] Yu X, Luo Y, Zhou Y (2008). BRCA1 overexpression sensitizes cancer cells to lovastatin via regulation of cyclin D1-CDK4-p21WAF1/CIP1 pathway: analyses using a breast cancer cell line and tumoral xenograft model. Int J Oncol.

[37] Sulaiman A, McGarry S, Li L (2018). Dual inhibition of Wnt and Yes-associated protein signaling retards the growth of triple-negative breast cancer in both mesenchymal and epithelial states. Mol Oncol.

[38] Göbel A, Browne AJ, Thiele S, Rauner M, Hofbauer LC, Rachner TD (2015). Potentiated suppression of Dickkopf-1 in breast cancer by combined administration of the mevalonate pathway inhibitors zoledronic acid and statins. Breast Cancer Res Treat.

[39] Oechsle CM, Showalter LE, Novak CM, Czerniecki BJ, Koski GK (2020). Statin drugs plus Th1 cytokines potentiate apoptosis and Ras delocalization in human breast cancer lines and combine with dendritic cell-based immunotherapy to suppress tumor growth in a mouse model of her-2(pos) disease. Vaccines (Basel).

[40] Zhang J, Li Q, Wu Y (2019). Cholesterol content in cell membrane maintains surface levels of ErbB2 and confers a therapeutic vulnerability in ErbB2-positive breast cancer. Cell Commun Signal.

[41] Liang Z, Li W, Liu J (2017). Simvastatin suppresses the DNA replication licensing factor MCM7 and inhibits the growth of tamoxifen-resistant breast cancer cells. Sci Rep.

[42] Lubet RA, Boring D, Steele VE, Ruppert JM, Juliana MM, Grubbs CJ (2009). Lack of efficacy of the statins atorvastatin and lovastatin in rodent mammary carcinogenesis. Cancer Prev Res (Phila).

[43] Kou X, Yang Y, Jiang X (2017). Vorinostat and simvastatin have synergistic effects on triple-negative breast cancer cells via abrogating Rab7 prenylation. Eur J Pharmacol.

[44] Kou X, Jiang X, Liu H (2018). Simvastatin functions as a heat shock protein 90 inhibitor against triple-negative breast cancer. Cancer Sci.

[45] Lin Z, Zhang Z, Jiang X (2017). Mevastatin blockade of autolysosome maturation stimulates LBH589-induced cell death in triple-negative breast cancer cells. Oncotarget.

[46] Xu P, Yu H, Zhang Z (2014). Hydrogen-bonded and reduction-responsive micelles loading atorvastatin for therapy of breast cancer metastasis. Biomaterials.

[47] Wolfe AR, Debeb BG, Lacerda L (2015). Simvastatin prevents triple-negative breast cancer metastasis in pre-clinical models through regulation of FOXO3a. Breast Cancer Res Treat.

[48] Howe EN, Burnette MD, Justice ME (2020). Rab11b-mediated integrin recycling promotes brain metastatic adaptation and outgrowth. Nat Commun.

[49] Mandal CC, Ghosh-Choudhury N, Yoneda T, Choudhury GG, Ghosh-Choudhury N (2011). Simvastatin prevents skeletal metastasis of breast cancer by an antagonistic interplay between p53 and CD44. J Biol Chem.

[50] Wang L, Wang Y, Chen A (2019). Pitavastatin slows tumor progression and alters urine-derived volatile organic compounds through the mevalonate pathway. FASEB J.

[51] Vintonenko N, Jais JP, Kassis N (2012). Transcriptome analysis and in vivo activity of fluvastatin versus zoledronic acid in a murine breast cancer metastasis model. Mol Pharmacol.

[52] Marti JL, Beckwitt CH, Clark AM, Wells A (2021). Atorvastatin facilitates chemotherapy effects in metastatic triple-negative breast cancer. Br J Cancer.

[53] Efimova EV, Ricco N, Labay E (2018). HMG-CoA reductase inhibition delays DNA repair and promotes senescence after tumor irradiation. Mol Cancer Ther.

